# Microbial Biofilms Along a Geochemical Gradient at the Shallow-Water Hydrothermal System of Vulcano Island, Mediterranean Sea

**DOI:** 10.3389/fmicb.2022.840205

**Published:** 2022-02-23

**Authors:** Valentina Sciutteri, Francesco Smedile, Salvatrice Vizzini, Antonio Mazzola, Costantino Vetriani

**Affiliations:** ^1^Department of Marine and Coastal Sciences, Rutgers, The State University of New Jersey, New Brunswick, NJ, United States; ^2^Department of Earth and Marine Sciences, University of Palermo, Palermo, Italy; ^3^Consorzio Nazionale Interuniversitario per le Scienze del Mare, Rome, Italy; ^4^Department of Biochemistry and Microbiology, Rutgers, The State University of New Jersey, New Brunswick, NJ, United States

**Keywords:** microbial biofilms, active microbial communities, ocean acidification, Vulcano island, sulfide oxidizing bacteria, shallow-water hydrothermal vents, Epsilonproteobacteria/*Campylobacteria*, Gammaproteobacteria

## Abstract

Shallow water hydrothermal vents represent highly dynamic environments where strong geochemical gradients can shape microbial communities. Recently, these systems are being widely used for investigating the effects of ocean acidification on biota as vent emissions can release high CO_2_ concentrations causing local pH reduction. However, other gas species, as well as trace elements and metals, are often released in association with CO_2_ and can potentially act as confounding factors. In this study, we evaluated the composition, diversity and inferred functional profiles of microbial biofilms in Levante Bay (Vulcano Island, Italy, Mediterranean Sea), a well-studied shallow-water hydrothermal vent system. We analyzed 16S rRNA transcripts from biofilms exposed to different intensity of hydrothermal activity, following a redox and pH gradient across the bay. We found that elevated CO_2_ concentrations causing low pH can affect the response of bacterial groups and taxa by either increasing or decreasing their relative abundance. H_2_S proved to be a highly selective factor shaping the composition and affecting the diversity of the community by selecting for sulfide-dependent, chemolithoautotrophic bacteria. The analysis of the 16S rRNA transcripts, along with the inferred functional profile of the communities, revealed a strong influence of H_2_S in the southern portion of the study area, and temporal succession affected the inferred abundance of genes for key metabolic pathways. Our results revealed that the composition of the microbial assemblages vary at very small spatial scales, mirroring the highly variable geochemical signature of vent emissions and cautioning for the use of these environments as models to investigate the effects of ocean acidification on microbial diversity.

## Introduction

Biofilms are typically defined as assemblages of microbial cells of either single or multiple species, enclosed in a gelatinous matrix adhering to living and inert surfaces (Costerton, 1999). The formation of biofilms is a multi-step process where nude surfaces are initially conditioned with organic and inorganic molecules forming a primary film, which successively attracts the microbial cells (Flemming and Wingender, 2010). After adhesion to the surface, these microorganisms start producing extracellular polymers (EPS) forming the amorphous matrix surrounding their cells (Flemming et al., 2007). A complex three-dimensional structure including multiple layers of microcolonies (microbial cells and EPS) separated by interstitial channels characterizes mature biofilms, which constitute heterogeneous and dynamic communities attached to the surfaces (Davey and O’toole, 2000). Biofilms represent the predominant form of microbial life in the marine environment, ranging from the surface to the deep ocean as well as in the water column where they constitute the precursor nucleus of marine snow (Dobretsov, 2010). In the photic zone, bacteria and microalgae are the main organisms constituting microbial biofilms which include also microscopic fungi, heterotrophic flagellates and sessile ciliates (Decho, 2000).

The importance of biofilms in the ecology of benthic ecosystems is widely recognized. Indeed, biofilms not only represent the main food source for a variety of grazers, but can actively control the development of benthic communities by influencing the settlement of algal spores and invertebrate larvae, including relevant aquaculture species such as *Mytilus galloprovincialis* (Bao et al., 2007; Hadfield, 2011). Biofilms also provide valuable ecosystem services including primary production, nutrient recycling, organic matter degradation and sediment trapping (Bhaskar and Bhosle, 2005; Ortega-Morales et al., 2010). Several compounds with potential application in biotechnology are isolated from microorganisms within biofilms, particularly from those living in extreme marine environments (Mancuso Nichols et al., 2005). On the other hand, biofilms colonizing artificial surfaces such as oil and gas installations, aquaculture nets and ship hulls alter the physical and chemical properties of these structures causing great economic losses in the maritime industry (Qian et al., 2007; Garrett et al., 2008; Salta et al., 2013). For all the reasons stated above, microbial biofilms are both ecologically and economically relevant.

In recent years, microbial biofilms have been investigated also in the context of climate change and its effects, including ocean acidification. In this regard, Lidbury et al. (2012) reported increased biomass as well as shifts in the assemblage of the community of biofilms along a natural *p*CO_2_/pH gradient in Levante Bay at Vulcano vents. In the same area, chlorophyll-*a* concentration in microphytobenthos communities were higher at low pH sites (pH: 7.9) compared to control sites (pH: 8.1), with changes in the composition of benthic diatom assemblages observed on both artificial (Johnson et al., 2013) as well as natural surfaces (Johnson et al., 2015). Although some changes in the community composition of biofilms could be explained by taxa specific response to acidification (Witt et al., 2011; Taylor et al., 2014), metabolic activity measured as oxygen fluxes (i.e., oxygen production and consumption rates) would not be affected by ocean acidification (Witt et al., 2011). More recently, Hassenrück et al. (2017) demonstrated that the diversity of mature biofilms in coral reef systems was scarcely influenced by pH changes, whereas other biotic and abiotic factors such as light exposure and grazing intensity controlled the biofilm community which, in turn, conditioned the settlement of coral larvae. Furthermore, sediment bacterial community composition showed variations in the abundance of few taxa in relation to long-term acidification at Vulcano vents, but overall the organisms appeared to persist under the acidified conditions (Kerfahi et al., 2014). Based on these observations, and the fact that pH variations naturally occurring in the aquatic environments are well tolerated by microorganisms, some authors argue that, overall, the degree of pH variations due to anthropogenic ocean acidification might not dramatically affect microbial communities (Joint et al., 2011). In order to investigate the response of microbial biofilms to ocean acidification, a colonization experiment was conducted in the shallow-water vent system of Levante Bay (Vulcano Island), a site considered as analog of future acidified oceans (Boatta et al., 2013; Aiuppa et al., 2021). The aim of this study was to evaluate the influence of hydrothermal vent emissions at Vulcano island on the diversity, composition and inferred functional profiles of microbial biofilms, based on the hypothesis that natural acidification induced by the elevated CO_2_ concentrations of vents emissions could affect the structure and functionality of these communities. We also aimed at evaluating the combined effect of CO_2_ and H_2_S emissions on the biofilm communities, as the latter is also present at detectable concentrations in the southern part of Levante Bay (a detailed description of the area is provided in the following section).

## Materials and Methods

### Study Area

**FIGURE 1:**
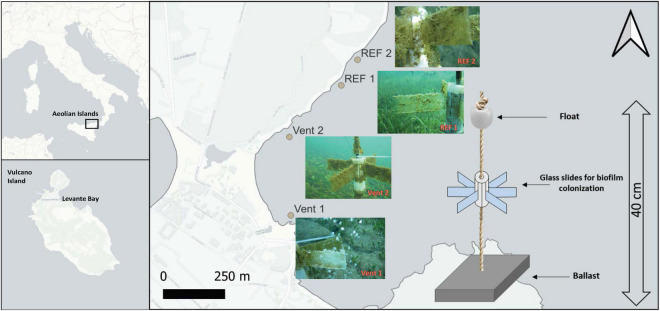
Map showing the location of the study site (Vulcano Island-Levante Bay) and the four sites chosen for the experiments. **(Right)** scheme of the satellite-like structure used for the experiment. **(Left)** pictures of the biofilms collected from the different sites at the end of the experiment.

Vulcano Island is part of the active volcanic arc of the Aeolian Islands archipelago in the Southern Tyrrhenian Sea (Mediterranean Sea, Figure 1). Since its last eruption (1888–1890), the volcano has been in a state of solfataric activity, characterized by the presence of both aerial as well as submerged fumaroles mainly releasing CO_2_ for a total of 482 t day^–1^ and, to a lesser extent, H_2_S and other gas species (Inguaggiato et al., 2012). In Levante Bay small fumarolic emissions occur offshore at shallow depths (<10 m). Volcanic emissions are visible as bubble trains rising from the sea-bottom and are dominated by CO_2_ (98–99% vol of CO_2_), for a total estimated value of 3.6 t day^–1^ of CO_2_ (Inguaggiato et al., 2012). In the southernmost point of Levante Bay, bubbling gas discharges are also characterized by a variable concentration of H_2_S (1.57–2.47 vol%, Carapezza et al., 2011) probably derived by alkaline hydrolysis of metal sulfides promoted by weakly acidic waters (Capaccioni et al., 2001). However, the concentration of H_2_S decreases with the distance from the vents and only a small portion of the gas enters into the aqueous phase where it oxidizes to sulfate due to the high O_2_ saturation recorded in the bay, particularly in the northern area (Boatta et al., 2013). Due to the intense venting in the southern part of the Levante Bay, a pH gradient (pH: 5.65–8.1) runs parallel to the north-eastern coast of the island, with pCO_2_ ranging from 3361.7 ± 2971.3 μatm to 424.6 ± 61.5 μatm (Boatta et al., 2013). After a preliminary survey assessing the physicochemical parameters of Levante Bay, including temperature, salinity, pH and ORP, four sites along a pCO_2_/pH gradient were selected as suitable stations to conduct a biofilm colonization experiment (Figure 1): Vent 1 (38°24′59.05′′N, 14°57′38.76′′E), located in the southern point and representing the main venting area of the bay, is characterized by intense CO_2_ and H_2_S gas fluxes; Vent 2 (38°25′9.40′′N, 14°57′42.14′′E), located about 330 m north of Vent 1, is not a vent *sensu stricto* but rather an area characterized by small and sparse CO_2_-dominated emissions; REF 1 (38°25′14.33′′N, 14°57′52.94′′E) and REF 2 (38°25′17.12′′N, 14°57′56.77′′E), located about 560 m and 760 m north of the main venting area respectively, represent reference sites where neither vent emissions nor their influence have been detected.

### Experimental Design and Sampling Procedure

The taxonomic composition of microbial biofilm communities along the Levante Bay *p*CO_2_/pH gradient was assessed via a colonization experiment that took place between October and December 2016. Physicochemical parameters including temperature, pH, Oxidation-Reduction Potential (ORP) and salinity were periodically recorded over the duration of the experiment using a multiparameter probe (model HI98194; HANNA Instruments, Woonsocket, RI, United States).

Sterile microscope glass slides were used as substrate for biofilm colonization. Slides were assembled into a satellite-like structure connected to a float and, at the lower end, to a dead weight to secure the full structure to the sea bottom (Figure 1). These substrates were deployed at a depth of 3 m at the four sites along the *p*CO_2_/pH gradient (Vent 1, Vent 2, REF 1, and REF 2). Three structures (*n* = 3 replicates) were collected by scuba divers from each site at two times: t1 (17 days after the deployment) and t2 (57 days after the deployment) corresponding to the end of the experiment. Immediately after collection, the glass slides were disassembled from the structures. Biofilms were removed from each glass slide with a sterile blade, stored in RNA Later (Thermofisher, Waltham, MA, United States), transferred to the laboratory and preserved at –20°C for further analyses.

### Analytical Methods

In order to assess the diversity, composition and inferred functional profiles of microbial biofilm communities from Levante Bay experiments, both molecular and bioinformatic analyses were performed.

#### RNA Extraction, cDNA Synthesis, Amplification of the 16S RNA and Sequencing

RNA was extracted from RNA Later-stored biofilms using a phenol:chloroform extraction protocol. Briefly, 850 μl of extraction buffer (50 mM Tris-HCl, 20 mM EDTA, 100 mM NaCl; pH 8.0) and 100 μl of lysozyme (100 mg/ml) were added to 0.5 g of biofilm sample. After incubation at 37°C for 30 min, the samples were supplemented with 5 μl of proteinase K (20 mg/ml) and incubated as previously. This mix was then supplemented with 50 μl of SDS (20%) and incubated in a water bath at 65°C for 1 h. RNA was extracted in a series of phenol:chloroform:isoamylalcohol (25:24:21, pH 4.3) and chloroform:isoamylalcohol (24:21) extractions. Overnight precipitation of the extracted supernatant was performed using 3M sodium acetate and isopropanol. The precipitated sample was washed twice with 70% ice cold ethanol and resuspended in ultrapure water. A DNAse treatment (TURBO DNAse kit, Invitrogen, Carlsbad, CA, United States) was performed according to the manufacturer’s instructions to remove any DNA carryover from the extracted samples. The resulting RNA was used as a template in a reverse transcription reaction generating cDNA (Invitrogen cDNA synthesis kit, Invitrogen, Carlsbad, CA, United States), following the specifications of the manufacturer. To evaluate the integrity of the cDNA, the product of the reverse transcription reaction was used as a template for the polymerase chain reaction amplification (PCR) of 16S rRNA transcripts using primers Bact 8F (5′-AGAGTTTGATCCTGGCTCAG-3′) and Univ 519R (5′-ATTACCGCGGCTGCTGG- 3′). The diversity of biofilm communities from Levante Bay was evaluated by amplifying the variable 4 (V4) region of 16S rRNA transcripts using the prokaryotic universal primers (515f 5′-GTG CCA GCM GCC GCG GTA A-3′ and 806r 5′-GGA CTA CVS GGGTAT CTA AT-3′; Caporaso et al., 2011), and the HotStarTaq Plus Master Mix Kit (Qiagen, Hilden, Germany), under the following conditions: 94°C for 3 min, followed by 30 cycles at 94°C for 30 s, 53°C for 40 s and 72°C for 1 min, and by a final elongation step at 72°C for 5 min. Multiple PCR reactions were combined to reduce potential bias. The total number of reads16S rRNA amplicons were sequenced using a PMG Ion Torrent platform at the Molecular Research LP facility (Shallowater, TX, United States). Sequences are available through the NCBI Short Read Archive database with accession number PRJNA789583. At the research facility, sequences were depleted of barcodes and primers, then sequences < 150 bp were removed along with sequences with ambiguous base calls and with homopolymer runs > 6 bp. The total number of reads for all samples after the chimera check was 2,095,467. The average number of reads per sample was 87,311.

#### Bioinformatics

The 16S rRNA sequence analysis was conducted using the QIIME 1.9 software package (Caporaso et al., 2010). Chimeric sequences were removed using ChimeraSlayer (Haas et al., 2011). Operational Taxonomic Units (OTUs) were picked at 97% similarity using the “pick_open_reference_otus.py.” Clustering of OTUs was performed using the Greengenes database (McDonald et al., 2012). Taxonomic classification of clustered OTUs was done using the Ribosomal Database Project Classifier against the Silva 123 Database (Quast et al., 2013; Glöckner et al., 2014; Yilmaz et al., 2014). Functional profiles of biofilms communities were predicted using the software Tax4Fun, a tool for functional community profiling based on 16S rRNA data (Aßhauer et al., 2015). Briefly, Tax4Fun uses the normalized taxonomic abundances to linearly combine 16S rRNA gene-based biodiversity structure to a precomputed genomic reference profiles for the prediction of the functional profile of the microbial community (see Aßhauer et al., 2015 for further details).

### Phylogenetic Analyses

Sequences of the V4 region of 16S rRNA genes of OTUs and 16S rRNA sequences of their closest cultured relatives (obtained by searching the NCBI non-redundant database by nucleotide BLAST search) were aligned using Clustal Omega (Sievers et al., 2011) within SEAVIEW (Galtier et al., 1996; Gouy et al., 2010). The resulting alignment was used to reconstruct phylogenetic trees using the Maximum Likelihood algorithm with PhyML (Guindon and Gascuel, 2003) using the general time reversible (GTR) model, aLRT scores calculated.

### Statistical Elaboration

Differences between sites and among times in the physicochemical variables, taxonomic composition (at the Phylum, Class, and Genus levels) and predicted functional profiles of biofilms were tested through Permutational Analysis of Variance (PERMANOVA). In all cases, a 4 × 2 orthogonal design with “Site” and “Time” set as fixed factors was used to conduct the analysis on square root transformed data. The effect of the interaction of the two factors “Site” and “Time” was tested as well. Pair-wise tests were performed on significant results. Square-root transformed data were used in order to construct resemblance matrices based on Euclidean distance for physicochemical variables and Bray–Curtis distance for the taxonomic composition. Gower distance was applied for elaboration of the predicted functional profiles (Gower, 1971; Anderson et al., 2008). All statistical elaborations were performed at both multivariate and univariate level. Similarities among 16S RNA transcripts was visualized using non-metric multidimensional scale (nMDS) coupled with Cluster analysis. A SIMPER test was conducted in order to understand the relative contribution of single OTUs to the observed difference between samples using PAST Paleontological Statistics V3.25^1^; after that, a BLASTn (megablast) search was performed against the NCBI-National Center for Biotechnology Information nucleotide database^2^ in order to identify the most similar prokaryotic-derived sequence entry on the first twelve taxa identified by the SIMPER test.

Diversity indexes (Taxa_S, Individuals, Dominance, Simpson, Shannon, Evenness, Margalef, Chao-1) were also calculated on the PAST Paleontological Statistics V3.25 software using a Bray Curtis similarity matrix obtained from the OTUs abundance table. The PRIMER + PERMANOVA v6 software package (PRIMER-E Ltd., Plymouth, United Kingdom, Anderson et al., 2008) was used to perform the statistical elaborations on the physicochemical variables, the community structure and diversity of microbial biofilms as well as their predicted functional profiles.

## Results

### Environmental Settings

**FIGURE 2:**
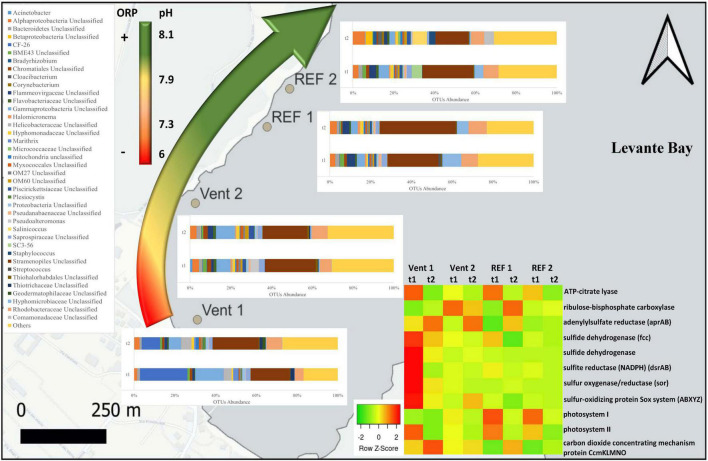
Genus-level taxonomic affiliation of 16S rRNA transcripts recovered from the biofilms located at the four study sites of Levante Bay, Vulcano island. Taxa representing genera that account for at least 1% (on average) of the overall abundance in all samples are shown. The curved arrow on the left indicates the redox (ORP) and pH observed along the *p*CO_2_/pH gradient. The heatmap on the right shows the abundance of genes for inferred central metabolic pathways of the biofilms.

Physicochemical parameters were measured at least once every 2 weeks throughout the experiment (Table 1). Temperature ranged from 18.3 to 22.7°C, showing similar values among sites and decreasing significantly over time (Supplementary Table S1). pH values, ranging from 6.7 to 8.3, showed significant differences between the vent and reference sites, as well as between Vent 1 and Vent 2 (Table 1 and Figure 2). ORP values ranged from –67.8 mV to 180.8 mV and showed a trend similar to the pH, with values increasing from Vent 1 toward the other sites, and significant differences over time. On the contrary, salinity remained relatively constant over time and across all sites (Table 1 and Figure 2).

**TABLE 1:** Physicochemical parameters of the study sites for the duration of the experiment.

	Vent 1	Vent 2	REF 1	REF 2
**October**				
**T [°C]**	22.74 ± 0.35	22.58 ± 0.08	22.75 ± 0.37	22.65 ± 0.23
**pH [unit]**	7.39 ± 0.49	8.03 ± 0.13	8.18 ± 0.04	8.28 ± 0.14
**ORP [mV]**	–49.68 ± 14.05	119.71 ± 25.94	145.66 ± 5.87	115.58 ± 7.08
**Sal.[psu]**	38.40 ± 0.00	38.38 ± 0.04	38.35 ± 0.14	38.41 ± 0.03
**November**				
**T [°C]**	21.02 ± 1.20	20.91 ± 1.14	20.88 ± 1.12	20.86 ± 1.09
**pH [unit]**	7.14 ± 0.46	7.88 ± 0.25	8.10 ± 0.09	8.14 ± 0.08
**ORP [mV]**	–32.99 ± 43.02	90.67 ± 76.05	73.38 ± 85.99	66.47 ± 78.79
**Sal.[psu]**	38.39 ± 0.06	38.39 ± 0.04	37.64 ± 1.36	38.22 ± 0.32
**December**				
**T [°C]**	18.51 ± 0.36	18.35 ± 0.61	18.28 ± 0.63	18.26 ± 0.63
**pH [unit]**	6.70 ± 0.77	7.89 ± 0.03	8.07 ± 0.04	8.15 ± 0.04
**ORP [mV]**	–11.51 ± 21.37	122.58 ± 48.50	164.71 ± 13.42	173.78 ± 10.01
**Sal.[psu]**	38.27 ± 0.01	38.27 ± 0.02	38.25 ± 0.06	38.28 ± 0.06

*Values are reported as mean ± standard deviation for each site in each month.*

### Community Structure and Diversity

In the biofilms collected from Levante Bay, up to >98% of the OTUs were affiliated to the domain Bacteria, while the remaining OTUs were associated to Archaea and unclassified sequences. Among the Archaea that could be classified, sequences related to *Nitrosopumilus* and *Methanobacterium* spp. were detected at very low abundances (<0.0025%). At the phylum level (Supplementary Figure S1A), the most abundant sequences across all samples were Proteobacteria (47.9%), Cyanobacteria (27.3%), and Bacteroidetes (11.8%). Multivariate analysis at this taxonomic level revealed, overall, significant differences among biofilms at Vent 1 *vs.* Vent 2, as well as Vent 1 *vs.* REF 1 (Supplementary Table S2).

On average at the class level (Supplementary Figure S1B), Gammaproteobacteria were numerically dominant at Vent 1 at the beginning of the experiment (45.8%) and were followed by chloroplast-related sequences (19.9%) and Alphaproteobacteria (8.1%). Other abundant groups in this site at t1 were Epsilonproteobacteria (aka *Campylobacteria*) and Deltaproteobacteria (4.8% and 3.7%, respectively). At the end of the experiment (t2), the abundance of Gammaproteobacteria in Vent 1 decreased considerably (22%), while Alphaproteobacteria doubled their abundance (16.5%); chloroplast-related sequences, Deltaproteobacteria and Epsilonproteobacteria slightly increased toward the end of the experiment (23.1, 5.7, 6.2% respectively at t2).

On average, the biofilm community of Vent 2 at t1 was dominated by chloroplast-related sequences and Gammaproteobacteria (25.5 and 23.5%, respectively), followed by Alpha- and Deltaproteobacteria (15.4 and 5.6%, respectively). After 57 days (t2), the community composition remained substantially similar, with a slight decrease in the relative abundance of chloroplast-related sequences and Gammaproteobacteria (22.5 and 22.9%, respectively), and a minor increase in the relative abundance of Alpha- and Deltaproteobacteria (17 and 6.2%, respectively). In REF 1, chloroplast-related 16S rRNA transcripts were always numerically dominant in the community, with their abundance increasing over time (from 26.1 to 38.1% for t1 and t2, respectively). Alphaproteobacteria were the second most abundant bacterial group at this site and remained relatively constant during the experiment (24.5 and 24.1% for t1 and t2, respectively).

The abundance of Gammaproteobacteria at REF 1 was lower than that of the other groups at this site, and lower than Gammaproteobacteria at both Vent sites. On average, Gammaproteobacteria decreased over the duration of the experiment (from 12.5% at t1, to 9.6% at t2). A similar trend was observed for Deltaproteobacteria (4.3 and 3.7% at t1 and t2, respectively). Cytophagia were numerically higher at this site compared to Vent 1 and Vent 2, although their abundance slightly decreased over time (from 5.2% at t1, to 3.9% at t2).

The composition of the biofilm community at REF 2 was similar to REF 1 at t1, with chloroplast-related sequences constituting 25.9% of the community, followed by Alphaproteobacteria (20.3% on average), Gammaproteobacteria (14.8%), Cytophagia (9.2%), and Deltaproteobacteria (4.1%). At the end of the experiment (t2), the relative average abundance of chloroplast-related sequences, Gammaproteobacteria, Cytophagia, and Deltaproteobacteria decreased (16.9, 11, 2.1, and 2.5%, respectively), whereas Alphaproteobacteria remained relatively constant (20.6%). Bacilli and Betaproteobacteria, which were scarcely present at t1, increased numerically in mature biofilms (t2), reaching an average of 10.3 and 10% of the overall community, respectively.

At the class level, the biofilm community of Vent 1 was significantly different compared to all the other sites, whereas biofilms of Vent 2 were significantly different compared to those of REF 1 (Supplementary Table S2).

At the genus level (Figure 2), sequences related to the *Thiothrix*-related group CF-26 were, on average, the most abundant (23.3%) in young biofilms (t1) at Vent 1; the other abundant groups were unclassified sequences of Stramenopiles and Gammaproteobacteria, representing respectively 19.3 and 14% of the total sequences, followed by unclassified taxa affiliated with the families Rhodobacteraceae and Helicobacteraceae (4.2 and 3.7%, respectively), and with the genus *Candidatus* Marithrix (2.4%). At the end of the experiment (t2), the relative abundance of these groups changed: the abundance of the *Thiothrix*-related group CF-26 decreased to 9.1%, while *Candidatus* Marithrix and unclassified sequences of Gammaproteobacteria decreased to 0.7 and 6%, respectively, and the relative abundance of unclassified Stramenopiles as well as of taxa affiliated with Rhodobacteraceae and Helicobacteraceae families increased (22.5, 7.6, and 5%, respectively).

At Vent 2, unclassified Stramenopiles constituted the 24.7% of the overall average abundance of young biofilms (t1). The most abundant genus belonging to the Gammaproteobacteria was *Pseudoalteromonas* (4% on average), while unclassified Gammaproteobacteria accounted for 6.9%. Within the Alphaproteobacteria, members of the Rhodobacteraceae accounted for 5.7%, while 3.2% remained unclassified. At the same site, the mature biofilm community (t2) showed a similar taxa assemblage, with the exception of *Pseudoalteromonas*-related sequences, whose abundance decreased drastically (<0.01%).

Chloroplast-related 16S rRNA transcripts from unclassified Stramenopiles constituted the major group at REF 1, with their abundance increasing from 25.1 to 37.4% over time. Sequences of unclassified Hyphomicrobiaceae and unclassified Rhodobacteraceae were also relatively abundant and showed different patterns, with the former decreasing (from 9.5 to 5.6%) and the latter remaining relatively constant during the experiment (from 8.1 to 8.3%).

The most abundant groups at the beginning of the experiment (t1) at REF 2 were unclassified Stramenopiles (24.8%) and unclassified Rhodobacteraceae (7.3%), followed by unclassified Gammaproteobacteria (5.1%), group SC3-56 of the phylum Bacteroidetes (5%) and unclassified Hyphomicrobiaceae (4.4%). At the end of the experiment, all these groups decreased in their abundance, while other taxa such as *Salinicoccus, Staphylococcus*, unclassified Alphaproteobacteria and unclassified Comamonadaceae increased greatly (6.9, 3.1, 5.9, and 4.8%, respectively).

Permanova analysis of the community composition at the genus level revealed significant differences for the factor “Site.” “Time” was also significant for the community structure, although to a lesser extent (Supplementary Table S2). Pair-wise tests revealed significant differences between Vent 1 vs. all the other sites for the factor “Site,” as well between Vent 2 and REF 1 (Supplementary Table S2).

**FIGURE 3:**
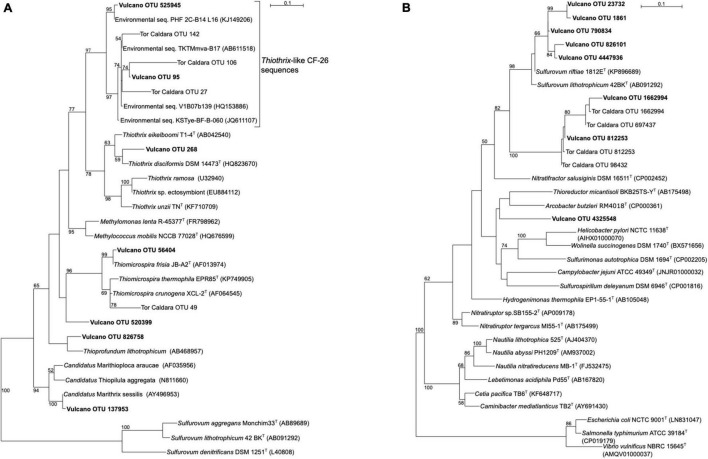
Maximum-likelihood phylogenetic trees derived from 16S rRNA transcript sequences from Vulcano. aLRT branch support values higher than 50% were based on 1000 replicates and are shown at each node. Bar, 0.1% substitutions per position. Tor Caldara sequences were reported in Patwardhan et al. (2018). **(A)** Phylogenetic tree showing sequences related to sulfide-oxidizing Gammaproteobacteria (in boldface). Sequences belonging to the class Epsilonproteobacteria were used as outgroup. **(B)** Phylogenetic tree showing sequences related to sulfide-oxidizing Epsilonproteobacteria (in boldface). Sequences belonging to the class Gammaproteobacteria were used as outgroup.

SIMPER analysis conducted on Bray-Curtis matrix highlighted the taxa that mostly contributed to the dissimilarity among the samples (Supplementary Table S3). The highest contribution (3.4%) was attributed to OTU ID 1836083, which was related OTU ID 95 and to uncultured Gammaproteobacteria belonging to the *Thiothrix*-related group CF-26 sequences, previously recovered from a shallow-water gas vent in the Mediterranean Sea (Tor Caldara OTU 27, Figure 3; Patwardhan et al., 2018) and other marine hydrothermal environments. The second taxa which contributed to 2.4% of the total dissimilarity was OTU ID 264, whose closest relative was an uncultured cyanobacterium retrieved from microbial biofilms from the Great Barrier Reef. Several sequences related to phototrophs, including Bacillariophyta as well as Foraminifera, contributed between 1.6 and 0.1% of the total dissimilarity. Interestingly, 1.2% of the sequences were related to halophilic bacteria such as *Salinicoccus* spp., hydrocarbon-degrading, sulfate-reducing Deltaproteobacteria (0.7%) as well as sulfide-oxidizing bacteria related to *Thiothrix* spp. (0.6%).

**FIGURE 4:**
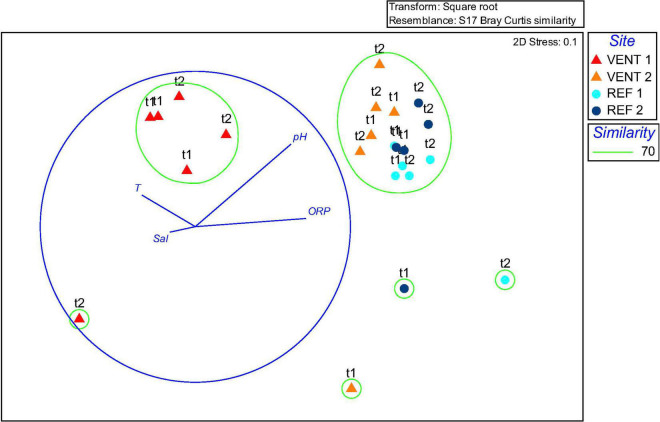
Non-metric multidimensional scaling (nMDS) plot of biofilms 16S rRNA transcript.

Similarity among communities from different sites at the genus level is shown by the nMDS plot (Figure 4), based on Bray-Curtis similarity calculated on the occurrence of sequences from the OTU table (n-MDS 2-D stress was 0.1). The nMDS ordination plot, coupled with Cluster analysis at 70% similarity level, showed the separation of two main groups: samples from Vent 1 formed a unique cluster, distinct from the majority of samples from the other sites which, in general, clustered in another group. Four samples (one from each site) clustered independently. The over-imposition of vectors representing environmental parameters showed that pH and ORP were responsible for the separation of Vent 1 from the other sites.

Diversity indexes showed similar values across sites and times with the exception of dominance and evenness, which differed significantly for the factor “Site.” Dominance was significantly different between Vent 1 and Vent 2 as well as between both Vent sites and REF 1. Evenness was significantly lower in Vent 1 compared to Vent 2 (see Supplementary Tables S4–S6 for detailed results).

### Predicted Functional Profiles of Vulcano Biofilm Communities

**FIGURE 5:**
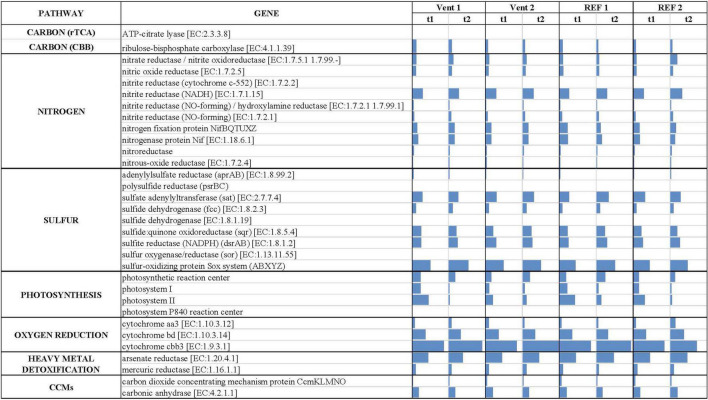
Predicted gene abundance (bars, normalized data) of key metabolic pathways in the biofilms collected from Levante Bay.

The inferred *in situ* expression of the central metabolic pathways in the marine biofilm communities collected in Levante Bay is reported in Figure 5 (numerical values in Supplementary Table S7). We reported the normalized abundances of inferred key genes involved in carbon fixation, nitrogen and sulfur metabolism, photosynthesis, oxygen reduction, heavy metals detoxification and carbon dioxide concentrating mechanisms (CCMs). Overall, genes for oxygen reduction, photosynthesis and sulfur metabolism were prevalent in the biofilm communities of Levante Bay. Multivariate analyses of each pathway revealed significant differences for carbon fixation cycles, sulfur metabolism, photosynthesis and CCMs only. **Carbon fixation:** the ATP-citrate lyase gene (encoding a diagnostic enzyme for the reductive tricarboxylic acid cycle) and the ribulose-bisphosphate carboxylase gene (RuBisCO, Calvin-Benson-Bassham cycle) were inferred to be significantly more prevalent in Vent 1 with respect to the other sites. However, RuBisCO decreased significantly over time (Supplementary Table S8). **Sulfur metabolism:** the genes for sulfur metabolism were inferred to be significantly more prevalent in the biofilms of Vent 1 compared to both REF sites at the beginning of the experiment (t1), while in mature biofilms (t2) significant changes occurred between Vent 1 and Vent 2 and REF 1, as well as between Vent 2 and REF 1. Time significantly affected the prevalence of genes involved in the sulfur metabolism in Vent 1 and REF 1. SIMPER analysis showed that the adenylylsulfate reductase (*aprAB*) gene contributed the most to the dissimilarity between Vent and REF sites, while the sulfate adenylyltransferase (*sat*) gene was the most determinant in the differences across time (Supplementary Tables S9, S10). **Photosynthesis:** the inferred prevalence of genes involved in photosynthesis was relatively high compared to the other metabolic pathways and showed significant changes in time (Supplementary Table S11). Indeed, the abundance of the two genes for photosystem II and photosystem I, which respectively contributed to the 49.7 and 34.7% of dissimilarity between t1 and t2, decreased significantly across time at all sites, while the genes for the photosystem P840 reaction center was inferred to be present only at Vent 1, t1. **Carbon concentrating mechanisms:** the genes for carbon dioxide concentrating mechanisms were inferred to be significantly lower in Vent 1 compared to both REF sites, as well as at t1 vs. t2 for the factor “Time” (Supplementary Table S11). The gene encoding for the carbon dioxide concentrating mechanism protein (*CcmKLMNO*) was responsible for the differences detected overall (Supplementary Table S11). **Nitrogen metabolism:** The gene encoding for the NADH-nitrite reductase [EC:1.7.1.15] was inferred to be the most abundant of the nitrogen metabolism genes in the biofilms communities of Levante Bay, followed by the gene encoding the nitrogenase complex (*Nif*; EC:1.18.6.1). The inferred abundance pattern for the nitrogen metabolism-related genes was similar across sites and times (Supplementary Table S7). **Oxygen reduction:** the gene encoding for the cytochrome cbb3 [EC:1.9.3.1] was inferred to be the most abundant of all the genes included in this analysis, followed by cytochrome bd encoding gene [EC:1.10.3.14] (Supplementary Table S7). **Heavy metal detoxification:** the arsenate reductase encoding gene [EC:1.20.4.1] was predicted to occur frequently, albeit with no significant changes overall.

## Discussion

Although microbial biofilms are key components of benthic communities and provide relevant ecosystem services in the marine environment, their response to ocean acidification is not clear. This study aimed at filling this gap by investigating the community composition of biofilms exposed to natural acidification in Levante Bay (Vulcano Island, Mediterranean Sea), a site considered analog for future acidified oceans. Based on previous studies of the Vulcano hydrothermal system, we hypothesized that acidification induced by elevated CO_2_ concentration might affect the abundance and/or the metabolic activity of photosynthetic microorganisms within the biofilms. Alternatively, we posited that the elevated concentration of H_2_S, serving as the main electron donor for the biofilm communities, might become the main driver of microbial metabolism and overcome the effect of CO_2_. In that case, we expected that sulfide-oxidizing bacteria would achieve a competitive advantage in the biofilm community.

Our findings showed that the distribution and abundance of few bacterial groups varied along the *p*CO_2_/pH gradient and identified significant changes in the diversity of the microbial communities and of their predicted functional profiles. These changes, however, were not always attributable exclusively to elevated concentration of CO_2_. Indeed, we found that factors related to hydrothermal vent activity in Levante Bay, such as the concentration of H_2_S (Boatta et al., 2013) and the consequent negative ORP values detected in the southern portion of the Bay (site Vent 1 in our study), significantly affected the composition and functionality of microbial biofilms by selecting microorganisms able to use sulfide an energy source. The lowest pH and ORP value were measured at Vent 1, the main venting area of the bay, in line with the elevated H_2_S and *p*CO_2_ values previously measured at this site (Capaccioni et al., 2001; Boatta et al., 2013).

### The Characteristics of the Vent 1 Biofilm Community Set It Apart From the Other Sites

Gammaproteobacteria were abundant in biofilms from Vent 1 and Epsilonproteobacteria were also detected, albeit to a lesser extent (Figure 2). Both these taxa include sulfide-oxidizing bacteria and are usually dominant in microbial communities of shallow and deep-sea hydrothermal vents. Gammaproteobacteria are usually more prevalent in lower sulfide habitats, while Epsilonproteobacteria dominate in higher sulfide habitats (Engel et al., 2004; Macalady et al., 2008; Reigstad et al., 2011; Giovannelli et al., 2013; O’Brien et al., 2015; Miranda et al., 2016; Meier et al., 2017; Patwardhan et al., 2018, 2021). Phylogenetic analyses of representative gammaproteobacterial sequences revealed that *Thiothrix*-, *Thiomicrospira*-, *Thioprofundum*-, and *Candidatus* Marithrix -related bacteria were active at Vent 1 (Figure 3A), while active members of the Epsilonproteobacteria were mainly related to *Sulfurovum* spp. (Figure 3B). These bacteria are chemolithoautotrophs that conserve energy by sulfide-oxidation (Jannasch et al., 1985; Lentini et al., 2014; Giovannelli et al., 2016; Patwardhan et al., 2021) suggesting that the oxidation of reduced sulfur species is one of the main energy-yielding process within the biofilm community at Vent 1. These observations are in line with the geochemical characteristics of Vent 1 (CO_2_ and H_2_S availability) and with the predicted metabolic pathways at this site, which revealed sulfide oxidation (mainly via the Sox, Fcc, and SQR pathways) and carbon fixation (via the CBB cycle) to be the main metabolisms (Supplementary Tables S7–S9). Within the Gammaproteobacteria, the relatively higher abundance of *Thiothrix*- and *Candidatus* Marithrix -related sequences compared to Epsilonproteobacteria (Figure 2) is likely due to the lower concentration of sulfide at Vent 1 compared to other geothermal habitats colonized by these organisms, such as deep-sea and shallow-water hydrothermal systems (Macalady et al., 2008; Giovannelli et al., 2013; O’Brien et al., 2015; Meier et al., 2017; Patwardhan et al., 2018, 2021). Interestingly, the gamma- and epsilonproteobacterial sequences from the Vulcano biofilms at Vent 1 were closely related to those retrieved from Tor Caldara, a coastal CO_2_ and H_2_S gas vent in the Mediterranean Sea (Figures 3A,B; Patwardhan et al., 2018). A metaproteogenomic study of the Tor Caldara biofilms demonstrated that *Thiothrix*- and *Thiomicrospira*-related Gammaproteobacteria, as well as *Sulfurovum*-related Epsilonproteobacteria, expressed pathways for sulfide oxidation and carbon fixation (Patwardhan et al., 2021), in line with the inferred metabolism of the Vulcano biofilms at the Vent 1 site (Figure 5). At Vent 1, biofilms were visually different compared to those from the other sites, and white and brown biofilms colonized the same glass slides and were very close to each other (Figure 1). Indeed, the biofilm community of Vent 1 formed a unique cluster, whose separation from the other biofilms was driven by pH and ORP (Figure 4). The simultaneous inputs of H_2_S and CO_2_ from hydrothermal vents causing low pH and negative ORP values at Vent 1 allowed the assemblage of a metabolically diverse community, where the distribution of organisms with different trophic strategies followed a geochemical gradient at a very small spatial scale. Indeed, the exposure to different environmental conditions was responsible for the co-existence of two communities on the same glass slide: the portion of slide directly exposed to the sulfide-rich hydrothermal emissions was covered with white biofilms of chemosynthetic sulfide-oxidizing Epsilon- and Gammaproteobacteria; the portion of the slide less directly exposed to the vent emissions was instead covered with brown biofilms likely composed of photo- and heterotrophic bacteria (Figure 1). These observations suggest that at the southern area of Levante Bay (Vent 1), where hydrothermal vents activity is particularly intense, high H_2_S emissions have a stronger effect on the composition of the microbial communities than CO_2_, which might cause bias in the interpretation of data relative to ocean acidification. At sites where H_2_S was undetectable and conditions more realistically simulate the ocean acidification scenario (e.g., Vent 2), the composition of the biofilm community shifted from chemosynthetic, sulfide-oxidizing Gammaproteobacteria to phototrophic and heterotrophic microorganisms (e.g., *Pseudoalteromonas* spp.; Figure 2).

### The Vulcano Hydrothermal System as Proxy to Study Ocean Acidification

The response of bacteria to natural acidification was highly variable in our study and for some groups partially in contrast with previous observations on intertidal biofilm communities from the same area (Taylor et al., 2014). These differences could be attributed to seasonal (autumn = this study; spring = Taylor et al., 2014), as well as habitat (subtidal = this study; intertidal = Taylor et al., 2014) effects, as microbial biofilms are dynamic complex entities and rapidly respond to environmental variations. In this study, sequences identified as Chloroplast (class-level, Supplementary Figure S1B) or unclassified Stramenopiles (genus-level, Figure 2) refer to Cyanobacteria and other oxygenic phototrophs, as the genomes of eukaryotic plastid contain Cyanobacteria-related 16S rRNA genes due to their prokaryotic origin (*sensu de* Lynn Margulis’ theory on Endosymbiotic Origin). Considering the high degree of conservation of the sequences, a more detailed taxonomic classification was not obtained as further analysis would be beyond the scope of this study. Diatoms, along with Cyanobacteria, have been indicated as potential “winners” in the future high-CO_2_ world, especially for the advantage they can gain by regulating the activity of their carbon concentrating mechanisms (Raven et al., 2012; Sandrini et al., 2014). Contrary to this expectation, the abundance of oxygenic phototrophs (unclassified Stramenopiles in Figure 2) did not increase in low pH conditions compared to the reference sites. Biofilms in reference sites showed an assemblage typical for marine biofilm communities, where Alphaproteobacteria (Rhodobacteraceae), Bacteroidetes (Cytophagia and Flavobacteria), Cyanobacteria and other phototrophs are usually abundant. These communities were relatively stable in their composition over time, except in one reference site (REF 2) where biofilms at the end of the experiment were characterized by elevated abundance of Bacilli, in particular *Salinicoccus* and *Staphylococcus* taxa, as well as members of Comamonadaceae family (Betaproteobacteria). Since these organisms typically inhabit sediments, wastewaters and activated sludge (Rosenberg, 2013), their presence may indicate that runoff from terrestrial sources might have occurred at this site before the end of the experiment, causing the shifts observed in the community over time.

### Predicted Functional Profiles

The analysis of the inferred functional profiles revealed differences in the abundance of functional genes in relation to both sites and times for carbon fixation, sulfur metabolism, photosynthesis, and CCMs. The predicted occurrence of RuBisCO, the key gene of the CBB cycle for carbon fixation, common both the sulfur-oxidizing Gammaproteobacteria and oxygenic phototrophs, along with genes for carbon fixation via the rTCA cycle and for sulfur metabolism (Figures 2, 5), characterized the functional profile of Vent 1 and reflected the abundance patterns of sulfur-oxidizing bacteria detected at this site such as *Thiothrix*, *Candidatus* Marithrix and *Thiothrix*-related group CF-26 and (Gammaproteobacteria) as well as *Sulfurovum spp*. (Epsilonproteobacteria; Figures 2, 3A,B). Genes for photosynthesis and the CBB cycle (RuBisCO) showed a decreasing pattern in their abundance over time (compare t1 and t2 at the various sites, Figures 2, 5). This trend suggests a time-dependent relative increase of heterotrophic bacteria over autotrophs in the biofilm communities of Levante Bay.

## Conclusion

In conclusion, this study showed shifts in the assemblage of biofilm community across the geochemical gradient of Levante Bay, Vulcano island. The effect of the emissions was particularly evident in the southern part of the bay (Vent 1) where the biofilm community included chemosynthetic, photo- and heterotrophic microorganisms. Here, the main environmental drivers were the low pH and the negative ORP resulting from the elevated concentrations of CO_2_ and H_2_S released from the vents, respectively (Figure 2 and Table 1). Their combined effect was evident on the diversity of the community which was characterized by higher dominance and lower evenness compared to the other sites. Following the pH gradient northwards in the bay, H_2_S concentrations decreased significantly while CO_2_ was still elevated resulting in positive ORP and low pH detected at Vent 2, respectively (Figure 2 and Table 1). The differences detected in the taxonomic composition were reflected in the inferred functionality of the biofilm communities, with increased abundance in genes for sulfur respiration in Vent 1 and Vent 2 compared to the reference sites (Figure 2). Although shallow-water vents are recognized as natural laboratories for testing the effects of ocean acidification on marine biota, the influence of other environmental variables cannot be excluded (Hassenrück et al., 2016; Dahms et al., 2018). Compared to previous studies conducted in the same area (Lidbury et al., 2012; Kerfahi et al., 2014; Taylor et al., 2014), our study highlights the importance of H_2_S in shaping marine biofilm communities in Levante Bay along with the effect of CO_2_. Further, acidification is likely to occur along with other environmental changes related to climate changes such as deoxygenation of the oceans (Keeling et al., 2010). In this regard, experimental investigations conducted at hydrothermal vents can provide insights on the response of microbial communities to multiple environmental stressors (i.e., elevated *p*CO_2_, low pH and low ORP) in the wide context of the global climate change scenario.

## Dedication

We dedicate this manuscript to the memory of Prof. Mario Giordano, dear mentor and colleague.

## Data Availability Statement

The datasets presented in this study can be found in online repositories. The names of the repository/repositories and accession number(s) can be found in the article/Supplementary Material.

## Author Contributions

VS, SV, AM, and CV designed the experimental procedure. VS collected the samples, performed the experiments, analyzed the data, and wrote the manuscript. SV and AM participated in the writing, review and editing of the manuscript. CV and FS participated to the data analysis and wrote the manuscript. All authors contributed to the article and approved the submitted version.

## Conflict of Interest

The authors declare that the research was conducted in the absence of any commercial or financial relationships that could be construed as a potential conflict of interest.

## Publisher’s Note

All claims expressed in this article are solely those of the authors and do not necessarily represent those of their affiliated organizations, or those of the publisher, the editors and the reviewers. Any product that may be evaluated in this article, or claim that may be made by its manufacturer, is not guaranteed or endorsed by the publisher.

## Footnotes

1
https://palaeoelectronica.org/2001_1/past/issue1_01.htm

2
https://www.ncbi.nlm.nih.gov/nucleotide/
