# Supplementary material for: Microbial Biofilms Along a Geochemical Gradient at the Shallow-Water Hydrothermal System of Vulcano Island, Mediterranean Sea
Source: Front Microbiol. 2022 Feb 23;13:840205. doi: 10.3389/fmicb.2022.840205 (PMC8905295; doi:10.3389/fmicb.2022.840205)
Supplement: Supplementary file 1 [file Data_Sheet_1.PDF]

Supplementary Material

1 Supplementary Data

Supplementary Material should be uploaded separately on submission. Please include any supplementary data, figures and/or tables. All supplementary files are deposited to FigShare for permanent storage and receive a DOI.

Supplementary material is not typeset so please ensure that all information is clearly presented, the appropriate caption is included in the file and not in the manuscript, and that the style conforms to the rest of the article. To avoid discrepancies between the published article and the supplementary material, please do not add the title, author list, affiliations or correspondence in the supplementary files.

2 Supplementary Figures and Tables

For more information on Supplementary Material and for details on the different file types accepted, please see here. Figures, tables, and images will be published under a Creative Commons CC-BY licence and permission must be obtained for use of copyrighted material from other sources (including re-published/adapted/modified/partial figures and images from the internet). It is the responsibility of the authors to acquire the licenses, to follow any citation instructions requested by third-party rights holders, and cover any supplementary charges.

2.1 Supplementary Figures

Supplementary Table 1. Permanova analysis at univariate level on the environmental parameters measured at the experimental sites (Vent 1, Vent 2, REF 1, REF 2) at three times (Oct = October; Nov = November; Dec = December). Significant results are highlighted. Pair-wise tests were conducted after significant P(perm) results.

	Temperature [°C]					pH [unit]					ORP [mV]					Salinity [psu]				
**Source**	**df**	**MS**	**Pseudo -F**	**P(perm)**	**Unique perms**	**df**	**MS**	**Pseudo -F**	**P(perm)**	**Unique perms**	**df**	**MS**	**Pseudo-F**	**P(perm)**	**Unique perms**	**df**	**MS**	**Pseudo-F**	**P(perm)**	**Unique perms**
**Site**	3	1,33E+02	4,67E+02	0,982	998	3	6,41E+01	2,08E+04	**0,001**	997	3	5,82E+04	1,21E+04	**0,001**	999	3	4,84E-01	4,08E-01	0,752	998
**Time**	2	12.621	4,43E+04	**0,001**	998	2	50	1,62E+04	0,201	999	2	1,56E+04	3,26E+03	**0,048**	998	2	5,62E-01	4,74E-01	0,69	998
**Site x Time**	6	2,57E+01	9,02E+00	1	997	6	1,71E+01	5,53E-01	0,77	999	6	2,50E-01	5,22E-01	0,799	998	6	4,70E-01	3,96E-01	0,834	999
**Residuals**	20	0,28495				20	30,887				20	4,79E-01				20	1,19E+04			
**Pair-wise tests**		**Groups**	**t**	**P(perm)**	**Unique perms**		**Groups**	**t**	**P(perm)**	**Unique perms**		**Groups**	**t**	**P(perm)**	**Unique perms**					
		Oct, Nov	4,08E+04	**0,003**	996		Vent 1, Vent 2	3,89E+04	**0,004**	993		Vent 1, Vent 2	5,24E+04	**0,001**	998					
		Oct, Dec	1,93E+04	**0,001**	984		Vent 1, REF 1	4,95E+04	**0,001**	995		Vent 1, REF 1	5,65E+04	**0,001**	995					
		Nov, Dec	5,77E+04	**0,001**	999		Vent 1, REF 2	5,26E+04	**0,001**	999		Vent 1, REF 2	5,69E+04	**0,001**	997					
							Vent 2, REF 1	2,30E+04	**0,043**	998		Vent 2, REF 1	4,91E-01	0,635	999					
							Vent 2, REF 2	3,09E+04	**0,007**	998		Vent 2, REF 2	2,31E-01	0,824	997					
							REF 1, REF 2	1,69E+04	0,119	996		REF 1, REF 2	2,75E-01	0,754	996					
												Oct, Nov	1,21E+00	0,248	998					
												Oct, Dec	2,63E+00	0,031	989					
												Nov, Dec	2,25E+00	**0,046**	995					

Supplementary Table 2. Permanova analysis at multivariate level on the community assemblages of biofilms. Pair-wise test showed comparison between sites (Vent 1, Vent 2, REF 1, REF 2) and times (t1 and t2). Significant results are highlighted. Pair-wise tests were conducted after significant P(perm) results.

	Philum					Class					Genus				
**Source**	**df**	**MS**	**Pseudo-F**	**P(perm)**	**Unique perms**	**df**	**MS**	**Pseudo-F**	**P(perm)**	**Unique perms**	**df**	**MS**	**Pseudo-F**	**P(perm)**	**Unique perms**
**Site**	3	415,71	2,2684	**0,007**	997	3	1056,4	3,1224	**0,001**	999	3	1987,8	30,139	**0,001**	999
**Time**	1	301,28	1,644	0,17	999	1	806,17	2,3829	0,055	997	1	1423,3	21,579	0,049	997
**Site x Time**	3	230,17	1,2559	0,251	998	3	375,41	1,1097	0,366	998	3	769,23	11,663	0,29	998
**Residuals**	16					16					16				

**Pair-wise tests Groups**	**t**	**P(perm)**	**Unique perms**	**t**	**P(perm)**	**Unique perms**	**t**	**P(perm)**	**Unique perms**
**Vent 1, Vent 2**	2,1857	**0,003**	984	2,4654	**0,004**	987	25748	**0,002**	985
**Vent 1, REF 1**	3,0676	**0,001**	986	3,5595	**0,001**	979	32537	**0,004**	986
**Vent 1, REF 2**	1,2473	0,231	988	1,7276	**0,02**	982	17742	**0,004**	986
**Vent 2, REF 1**	1,5517	0,063	983	1,902	**0,003**	989	1763	**0,006**	988
**Vent 2, REF 2**	1,1193	0,313	984	1,2403	0,211	987	10777	0,351	992
**REF 1, REF 2**	1,1841	0,277	989	1,2193	0,25	981	10724	0,354	983

Supplementary Table 3. SIMPER analysis coupled with BLASTn (megablast) search against NCBI-NUCLEOTIDE DATABASE showing the relative contribution of 16S rRNA sequences to the dissimilarity among samples.

OTU ID	SIMPER TEST		BLASTn (megablast) search against NCBI-NUCLEOTIDE DATABASE							
	**Av. dissim**	**Contrib. %**	**Accession**	**Best BLAST hit**	**E value**	**Perc. Ident.**	**Reference**	**Isolation source**	**Phylogenetic ascription**	**Closest relative**
1836083	2,696	3,792	AB611518.1	Uncultured gamma proteobacterium TKTMmva-B17	8E-134	98.90%	https://doi.org/10.1128/AEM.06491-11	Hydrothermal vents	Gammaproteobacteria	uncultured bacterium
264	1,729	2,432	JF261956.1	Uncultured bacterium clone Del10081A12	4E-132	98.53%	https://doi.org/10.1111/j.1574-6968.2011.02374.x	Marine biofilms from Great Barrier Reef	Bacteria; environmental samples	uncultured bacterium
428807	1,495	2,102	HM485054.1	Uncultured marine organism clone LON_240	4E-137	99.63%	10.1016/j.syapm.2011.01.003	Marine biofilms from Great Barrier Reef	Bacteria; environmental samples	uncultured marine organism
795504	1,109	1,56	HQ189720.1	Uncultured phototrophic eukaryote clone 40D06 chloroplast	4E-137	99.63%	10.3389/fmicb.2015.00818	Sea anemone Nematostella vectensis	Eukaryota; environmental samples	uncultured phototrophic eukaryote
			KY498709.1	*Angulodiscorbis quadrangularis* isolate AQ159-89 chloroplast	2E-135	99.27%	https://doi.org/10.1002/ece3.4358	Intertidal rocky shore	Eukaryota; Sar; Rhizaria; Retaria; Foraminifera; Rotaliida; Discorboidea; Glabratellidae; Angulodiscorbis	*Angulodiscorbis quadrangularis*
583758	1,017	1,43	MT229147.1	Uncultured bacterium clone 660336	7E-135	99.26%	10.1016/j.scitotenv.2020.141399	Hydrocarbon degrading bacteria	Bacteria; environmental samples	uncultured bacterium
			KP792487.1	Bacillariophyta sp. 867-32 plastid	8E-134	98.90%	doi.org/10.3389/fmars.2020.00585	Rocky-shore benthic foraminifera	Eukaryota; Sar; Stramenopiles; Ochrophyta; Bacillariophyta	Bacillariophyta sp. 867-32
			KP792475.1	Planoglabratella opercularis isolate GO_m22-06 plastid	8E-134	98.90%	doi.org/10.3389/fmars.2020.00586	Rocky-shore benthic foraminifera	Eukaryota; Sar; Rhizaria; Retaria; Foraminifera; Rotaliida; Discorboidea; Glabratellidae; Planoglabratella	*Planoglabratella opercularis*
517923	0,8387	1,18	FJ844231.1	Uncultured Firmicutes bacterium clone Fi07-21	4E-137	99.63%	10.1371/journal.pone.0027597	Salinity gradient	Bacteria; Firmicutes; environmental samples	uncultured bacterium

			LC164834.2	*Salinicoccus sp.* W19 gene	2E-135	99.27%	unpubl	Hypersalin environment	Bacteria; Firmicutes; Bacilli; Bacillales; Staphylococcaceae	*Salinicoccus sp.*
361839	0,8379	1,179	MH818895.1	Uncultured diatom clone H1716-16S_40 chloroplast	2E-135	99.27%	unpubl	Intertidal foraminifers	Eukaryota; Sar; Stramenopiles; Ochrophyta; Bacillariophyta	uncultured diatom
			EU182114.2	Uncultured bacterium clone D15_1_SW_H_48	2E-135	99.27%	10.1371/journal.pone.0066968	Bacterial communities from coastal areas	Eukaryota; Sar; Stramenopiles; Ochrophyta; Bacillariophyta	uncultured diatom
317794	0,7385	1,039	JF261956.1	Uncultured bacterium clone Del10081A12 16S	8E-134	98.90%	10.1111/j.1574-6968.2011.02374.x	Marine biofilms from the Great Barrier Reef	Bacteria; environmental samples	uncultured bacterium
New.ReferenceOTU145	0,6678	0,9394	MH818927.1	Uncultured diatom clone H1724-16S_26 chloroplast	9E-119	95.60%	unpubl	Intertidal foraminifers	Eukaryota; Sar; Stramenopiles; Ochrophyta; Bacillariophyta	uncultured diatom
			KC509521.1	*Coscinodiscus radiatus* chloroplast, complete genome	9E-119	95.60%	10.1093/gbe/evu039	Diatoms plastid genomes	Eukaryota; Sar; Stramenopiles; Ochrophyta; Bacillariophyta; Coscinodiscophyceae; Coscinodiscophycidae; Coscinodiscales; Coscinodiscaceae	*Coscinodiscus radiatus*
315090	0,5213	0,7333	GU118611.1	Uncultured bacterium clone Mfav_A15	9E-129	97.80%	10.1371/journal.pone.0009554	Caribbean Corals	Bacteria; environmental samples	uncultured bacterium
New.ReferenceOTU746	0,4682	0,6586	LC432074.1	Uncultured bacterium gene clone: GoM_AsphSed_Bac69	4E-122	96.34%	DOI:10.1007/978-3-030-34827-4_5	Asphaltic hydrocarbon emissions	Bacteria; environmental samples	hydrocarbon-degrading sulfate-reducing Deltaproteobacteria
New.ReferenceOTU772	0,4558	0,6412	KY276598.1	Uncultured bacterium clone CP_Otu1209	5E-126	99.60%	unpubl	Cochlodinium polykrikoides blooms (dinoflagellate)	Bacteria; environmental samples.	uncultured bacterium
			KC934138.1	Uncultured *Thiothrix sp.* clone PY10-10-194	4E-112	94.14%	10.1007/s11274-011-0812-5	Poyang Lake (China, freshwater)	Bacteria; Proteobacteria; Gammaproteobacteria; Thiotrichales; Thiotrichaceae; Thiothrix	uncultured *Thiothrix sp.*
			KF945141.1	Uncultured *Thiothrix sp.* clone DZ-8	4E-112	94.14%	unpubl	Activated sludge	Bacteria; Proteobacteria; Gammaproteobacteria; Thiotrichales; Thiotrichaceae; Thiothrix	uncultured *Thiothrix sp.*

Supplementary Table 4. Diversity Indexes of the biofilm samples across the four experimental sites (Vent 1, Vent 2, REF 1, REF 2) and times (t1 and t2). Values are reported as mean ± standard deviation.

Site	Time	Taxa_S		Individuals		Dominance_D		Simpson_1-D		Shannon_H		Evenness_e^H/S		Margalef		Chao-1	
		**mean**	**sd(±)**	**mean**	**sd(±)**	**mean**	**sd(±)**	**mean**	**sd(±)**	**mean**	**sd(±)**	**mean**	**sd(±)**	**mean**	**sd(±)**	**mean**	**sd(±)**
**Vent 1**		**6333,83**	**3073,78**	**99139,50**	**49401,81**	**0,03**	**0,01**	**0,97**	**0,01**	**5,62**	**0,38**	**0,06**	**0,05**	**546,08**	**248,73**	**10655,17**	**4925,05**
	**t1**	6959,00	2980,93	101243,67	53269,88	0,04	0,01	0,96	0,01	5,44	0,09	0,04	0,03	601,50	233,33	12154,67	4913,71
	**t2**	5708,67	3682,64	97035,33	57012,15	0,02	0,01	0,98	0,01	5,81	0,50	0,09	0,06	490,67	301,68	9155,67	5454,37
**Vent 2**		**7222,00**	**3740,03**	**86342,67**	**49272,45**	**0,01**	**0,00**	**0,99**	**0,00**	**6,79**	**0,44**	**0,15**	**0,07**	**632,92**	**306,61**	**11837,00**	**6084,06**
	**t1**	8446,67	5360,48	93084,33	47681,10	0,01	0,00	0,99	0,00	6,61	0,56	0,12	0,05	729,07	446,63	13988,33	8611,27
	**t2**	5997,33	1317,37	79601,00	60494,61	0,00	0,00	1,00	0,00	6,98	0,27	0,19	0,07	536,77	88,35	9685,67	2121,51
**REF 1**		**8479,17**	**4422,69**	**102029,50**	**47877,12**	**0,01**	**0,00**	**0,99**	**0,00**	**6,49**	**0,50**	**0,10**	**0,05**	**731,43**	**358,48**	**13831,00**	**6870,98**
	**t1**	10359,33	4885,00	103621,67	63772,89	0,01	0,00	0,99	0,00	6,87	0,27	0,12	0,07	894,30	379,20	16447,33	7811,83
	**t2**	6599,00	3798,95	100437,33	40693,50	0,02	0,00	0,98	0,00	6,12	0,33	0,08	0,03	568,57	312,90	11214,67	6038,69
**REF 2**		**6186,00**	**3965,22**	**69518,00**	**32925,01**	**0,02**	**0,02**	**0,98**	**0,02**	**6,14**	**1,33**	**0,11**	**0,05**	**546,66**	**337,76**	**9909,97**	**6536,39**
	**t1**	7928,00	4024,89	82573,00	31716,27	0,01	0,01	0,99	0,01	6,64	0,60	0,11	0,04	695,20	336,49	13038,00	6493,51
	**t2**	4444,00	3742,19	56463,00	34538,66	0,03	0,03	0,97	0,03	5,63	1,81	0,11	0,07	398,12	325,26	6781,93	5940,68

Supplementary Table 5. Permanova analysis on Dominance and Evenness diversity indexes calculated for the biofilm community. Significant results are highlighted. Pair-wise tests were conducted after significant P(perm) results.

	**Dominance**					**Evenness**			
**Source**	**df**	**MS**	**Pseudo-F**	**P(perm)**	**Unique perms**	**MS**	**Pseudo-F**	**P(perm)**	**Unique perms**
**Site**	3	918,35	5,3794	**0,009**	998	600,77	3,8492	**0,024**	999
**Time**	1	103,16	0,60426	0,5	999	138,77	0,88916	0,345	999
**Site x Time**	3	482,71	2,8276	0,053	999	194,38	1,2454	0,325	999
**Residuals**	16					156,07			

**Pair-wise tests Groups**	**t**	**P(perm)**	**Unique perms**	**t**	**P(perm)**	**Unique perms**
**Vent 1, Vent 2**	6,8914	**0,003**	985	3,0848	**0,029**	985
**Vent 1, REF 1**	3,3849	**0,015**	994	1,6732	0,127	987
**Vent 1, REF 2**	1,3987	0,169	987	2,1255	0,066	978
**Vent 2, REF 1**	3,7637	**0,011**	984	1,7293	0,115	985
**Vent 2, REF 2**	1,6714	0,114	977	1,1046	0,308	986
**REF 1, REF 2**	0,46971	0,769	986	0,57976	0,573	987

Supplementary Table 6. Permanova analysis on diversity indexes calculated for the biofilm community.

		**Taxa_S**				**Individuals**				**Simpson**			
**Source**	**df**	**MS**	**Pseudo-F**	**P(perm)**	**Unique perms**	**MS**	**Pseudo-F**	**P(perm)**	**Unique perms**	**MS**	**Pseudo-F**	**P(perm)**	**Unique perms**
**Site**	3	131,23	0,36	0,85	999	90,38	0,33	0,81	998	0,33	2,72	0,05	999
**Time**	1	583,39	1,61	0,22	999	90,54	0,34	0,56	997	0,01	0,05	0,85	998
**Site x Time**	3	110,89	0,31	0,89	998	53,20	0,20	0,91	999	0,29	2,39	0,08	999
**Residuals**	16	363,14				269,92				0,12			

		**Shannon**				**Margalef**				**Chao-1**			
**Source**	**df**	**MS**	**Pseudo-F**	**P(perm)**	**Unique perms**	**MS**	**Pseudo-F**	**P(perm)**	**Unique perms**	**MS**	**Pseudo-F**	**P(perm)**	**Unique perms**
**Site**	3	26,31	2,09	0,12	998	124,43	0,39	0,84	998	164,88	0,46	0,78	999
**Time**	1	9,12	0,72	0,45	998	567,93	1,77	0,20	999	662,15	1,86	0,17	999
**Site x Time**	3	16,26	1,29	0,31	998	104,34	0,33	0,87	995	138,50	0,39	0,87	999
**Residuals**	16	12,59				320,75				356,44			

Supplementary Table 7. Predicted gene abundance of selected metabolic pathways from biofilms collected at Levante Bay. Values are reported as mean ± standard deviation for each site (Vent 1, Vent 2, REF 1, REF 2) and time (t1, t2).

		Vent 1				Vent 2				REF 1				REF 2			
		t1		t2		t1		t2		t1		t2		t1		t2	
**PATHWAY**	**GENE**	**mean**	**sd (±)**	**mean**	**sd (±)**	**mean**	**sd (±)**	**mean**	**sd (±)**	**mean**	**sd (±)**	**mean**	**sd (±)**	**mean**	**sd (±)**	**mean**	**sd (±)**
**CARBON (r-TCA)**	ATP-citrate lyase [EC:2.3.3.8]	3,47E-06	2,31E-11	9,46E-07	2,51E-12	4,62E-07	2,79E-13	6,81E-08	1,55E-14	1,08E-07	2,28E-14	3,36E-07	1,24E-13	9,99E-08	1,52E-14	1,41E-07	9,50E-14
**CARBON (CBB)**	ribulose-bisphosphate carboxylase [EC:4.1.1.39]	1,74E-04	2,07E-09	1,32E-04	7,75E-09	1,11E-04	8,52E-10	1,02E-04	3,28E-10	1,31E-04	3,09E-10	6,42E-05	7,63E-11	1,02E-04	1,28E-09	9,44E-05	1,36E-09
**NITROGEN**	nitrate reductase / nitrite oxidoreductase [EC:1.7.5.1 1.7.99.-]	1,85E-04	1,33E-09	2,04E-04	4,03E-09	1,54E-04	1,41E-09	1,72E-04	3,28E-09	1,52E-04	1,53E-10	1,22E-04	2,23E-10	1,46E-04	4,81E-10	3,03E-04	3,73E-08
	nitric oxide reductase [EC:1.7.2.5]	1,65E-04	1,08E-10	1,46E-04	4,59E-09	1,27E-04	3,29E-09	1,41E-04	5,61E-10	1,67E-04	2,12E-10	1,29E-04	9,70E-10	1,44E-04	3,84E-09	1,09E-04	9,32E-09
	nitrite reductase (cytochrome c-552) [EC:1.7.2.2]	1,51E-06	2,36E-13	4,90E-07	5,54E-13	3,25E-06	1,24E-12	3,26E-06	5,61E-12	4,02E-06	3,91E-13	2,17E-06	6,88E-13	2,29E-06	2,02E-12	3,84E-06	2,28E-11
	nitrite reductase (NADH) [EC:1.7.1.15]	4,69E-04	1,69E-09	4,68E-04	3,34E-10	4,83E-04	1,02E-09	5,03E-04	1,98E-10	4,26E-04	1,68E-09	4,82E-04	6,76E-10	4,84E-04	4,00E-09	5,38E-04	3,55E-09
	nitrite reductase (NO-forming) / hydroxylamine reductase [EC:1.7.2.1 1.7.99.1]	3,05E-05	6,10E-11	1,21E-05	1,03E-11	2,30E-05	1,53E-10	3,13E-05	2,69E-10	2,00E-05	2,07E-11	9,63E-06	6,72E-11	2,37E-05	4,11E-11	1,33E-05	1,62E-10
	nitrite reductase (NO-forming) [EC:1.7.2.1]	7,42E-05	4,29E-10	9,92E-05	1,55E-10	8,59E-05	1,91E-10	9,60E-05	1,82E-10	1,04E-04	7,25E-12	9,68E-05	7,29E-10	8,87E-05	1,83E-09	1,09E-04	8,74E-09
	nitrogen fixation protein NifBQTUXZ	2,14E-04	2,01E-09	2,62E-04	2,31E-08	2,61E-04	5,60E-09	2,52E-04	2,68E-10	3,65E-04	4,93E-09	1,94E-04	7,08E-10	2,78E-04	1,64E-08	2,64E-04	8,07E-09
	nitrogenase protein Nif [EC:1.18.6.1]	2,67E-04	2,04E-09	2,58E-04	7,98E-09	2,56E-04	1,58E-08	2,66E-04	3,33E-09	3,74E-04	8,73E-10	2,45E-04	5,36E-10	2,98E-04	2,73E-08	2,00E-04	3,75E-08
	nitroreductase	5,62E-05	1,34E-11	3,54E-05	4,43E-10	4,21E-05	4,70E-10	4,91E-05	1,88E-10	3,91E-05	6,47E-10	3,14E-05	1,67E-10	4,40E-05	8,28E-10	5,17E-05	2,35E-09
	nitrous-oxide reductase [EC:1.7.2.4]	2,45E-05	7,51E-11	1,30E-05	3,33E-11	2,87E-05	1,33E-10	2,20E-05	3,36E-11	1,91E-05	1,42E-10	1,13E-05	1,62E-12	1,47E-05	1,33E-14	1,26E-05	7,41E-12
**SULFUR**	adenylylsulfate reductase (aprAB) [EC:1.8.99.2]	3,80E-05	2,05E-10	1,67E-05	2,64E-11	1,12E-05	1,88E-10	2,20E-05	2,81E-12	1,14E-05	2,29E-11	3,63E-06	2,45E-11	1,03E-05	1,04E-11	9,29E-06	1,31E-10
	polysulfide reductase (psrBC)	4,14E-07	8,47E-14	2,28E-07	1,41E-13	6,11E-07	1,36E-13	1,21E-06	1,09E-12	1,77E-06	2,59E-14	9,64E-07	8,03E-15	1,41E-06	4,89E-13	1,89E-06	8,52E-12

**SULFUR**	sulfate adenylyltransferase (sat) [EC:2.7.7.4]	4,59E-04	1,63E-09	4,31E-04	6,43E-09	5,01E-04	1,00E-08	5,14E-04	7,36E-10	4,25E-04	1,36E-09	5,54E-04	6,47E-09	5,27E-04	3,27E-08	4,74E-04	1,63E-08
	sulfide dehydrogenase [flavocytochrome c] flavoprotein chain [EC:1.8.2.3]	1,57E-04	3,40E-10	1,79E-04	1,74E-10	1,45E-04	6,18E-10	1,78E-04	6,76E-10	1,24E-04	5,69E-10	1,63E-04	7,77E-11	1,37E-04	4,65E-11	1,41E-04	2,76E-09
	sulfide dehydrogenase [EC:1.8.1.19]	2,37E-07	2,50E-13	4,13E-09	1,03E-16	3,88E-08	3,95E-15	2,38E-08	3,40E-15	7,05E-08	8,34E-16	2,41E-08	3,48E-15	3,63E-08	4,17E-15	1,42E-08	1,20E-15
	sulfide:quinone oxidoreductase [EC:1.8.5.4]	4,07E-04	1,73E-10	3,42E-04	1,35E-09	3,44E-04	3,74E-09	3,96E-04	2,43E-09	3,56E-04	2,59E-09	3,83E-04	1,68E-09	3,59E-04	1,72E-09	3,18E-04	2,38E-08
	sulfite reductase (NADPH) (dsrAB) [EC:1.8.1.2]	4,05E-04	8,68E-11	4,22E-04	2,56E-10	4,76E-04	7,22E-09	4,53E-04	1,48E-09	4,09E-04	8,42E-10	4,77E-04	2,23E-09	4,24E-04	5,22E-11	4,34E-04	2,06E-09
	sulfur oxygenase/reductase [EC:1.13.11.55]	1,75E-06	2,17E-13	3,68E-07	2,12E-13	2,59E-08	4,03E-15	2,00E-07	6,65E-14	5,47E-09	1,79E-16	0,00E+00	0,00E+00	0,00E+00	0,00E+00	0,00E+00	0,00E+00
	sulfur-oxidizing protein Sox system (ABXYZ)	8,53E-04	1,46E-09	9,21E-04	5,41E-09	8,25E-04	3,76E-09	8,45E-04	1,18E-10	7,44E-04	9,92E-09	8,70E-04	4,02E-10	7,42E-04	2,43E-08	7,89E-04	1,94E-08
**PHOTOSYNTHTESIS**	photosynthetic reaction center	3,62E-04	9,41E-09	2,87E-04	3,18E-09	2,50E-04	2,30E-08	3,23E-04	1,08E-08	2,91E-04	1,09E-09	3,91E-04	6,11E-10	2,48E-04	2,15E-08	2,14E-04	6,97E-08
	photosystem I	3,76E-04	1,83E-08	1,49E-05	3,33E-10	1,61E-04	7,92E-09	8,26E-05	5,78E-09	3,42E-04	6,35E-09	9,48E-05	6,79E-09	2,44E-04	3,67E-09	3,10E-05	2,11E-09
	photosystem II	7,54E-04	6,50E-08	2,99E-05	1,35E-09	3,36E-04	3,42E-08	1,70E-04	2,45E-08	7,05E-04	2,96E-08	2,04E-04	3,20E-08	5,09E-04	1,65E-08	6,50E-05	9,26E-09
	photosystem P840 reaction center	4,26E-06	1,09E-10	0,00E+00	0,00E+00	0,00E+00	0,00E+00	0,00E+00	0,00E+00	0,00E+00	0,00E+00	0,00E+00	0,00E+00	0,00E+00	0,00E+00	0,00E+00	0,00E+00
**OXYGEN REDUCTION**	cytochrome aa3 [EC:1.10.3.12]	1,07E-04	1,66E-10	1,19E-04	1,41E-09	1,30E-04	1,15E-08	7,42E-05	2,05E-11	8,18E-05	4,59E-11	7,74E-05	4,72E-10	1,07E-04	4,43E-09	2,20E-04	8,69E-08
	cytochrome bd [EC:1.10.3.14]	6,13E-04	1,16E-11	5,48E-04	5,77E-09	5,98E-04	4,42E-08	5,66E-04	8,29E-09	5,92E-04	5,24E-10	5,31E-04	3,39E-09	5,77E-04	3,87E-09	6,15E-04	4,98E-08
	cytochrome cbb3 [EC:1.9.3.1]	1,48E-03	3,78E-09	1,54E-03	2,49E-08	1,44E-03	1,54E-07	1,67E-03	3,55E-09	1,45E-03	6,60E-09	1,61E-03	1,62E-08	1,43E-03	4,08E-08	1,22E-03	5,99E-07
**HEAVY METAL DETOXIFICATION**	arsenate reductase [EC:1.20.4.1]	7,42E-04	1,63E-09	6,47E-04	4,59E-10	7,63E-04	3,12E-09	7,60E-04	1,37E-10	7,64E-04	1,30E-09	7,97E-04	6,87E-10	7,67E-04	4,54E-10	7,70E-04	5,14E-08
	mercuric reductase [EC:1.16.1.1]	1,34E-04	3,16E-11	1,23E-04	8,01E-10	1,43E-04	1,72E-09	1,65E-04	1,74E-09	1,58E-04	4,28E-10	1,06E-04	8,71E-10	1,64E-04	1,45E-09	1,51E-04	2,72E-09
**CCMs**	carbon dioxide concentrating mechanism protein CcmKLMNO	1,90E-05	1,55E-10	1,08E-05	3,53E-10	4,37E-05	2,03E-09	2,61E-05	8,58E-12	8,44E-05	2,62E-10	3,04E-05	2,52E-10	7,74E-05	3,11E-10	3,11E-05	1,82E-10
	carbonic anhydrase [EC:4.2.1.1]	2,87E-04	5,19E-10	2,92E-04	4,13E-09	3,26E-04	2,10E-09	2,95E-04	8,82E-10	3,30E-04	5,55E-09	2,78E-04	9,14E-10	3,07E-04	7,62E-11	3,24E-04	6,10E-09

Supplementary Table 8. Permanova analysis at univariate level of key metabolic pathways. Pair-wise test showed comparison between sites (Vent 1, Vent 2, REF 1, REF 2) and times (t1 and t2). Significant results are highlighted. Pair-wise tests were conducted after significant P(perm) results.

		Nitrogen				Oxygen Reduction				Heavy Metals Detoxification			
**Source**	**df**	**MS**	**Pseudo-F**	**P(perm)**	**Unique perms**	**MS**	**Pseudo-F**	**P(perm)**	**Unique perms**	**MS**	**Pseudo-F**	**P(perm)**	**Unique perms**
**Site**	3	37,968	1,0802	0,38	997	27,183	0,97456	0,459	999	15,885	2,5704	0,063	999
**Time**	1	86,025	2,4475	0,06	999	2,7863	0,099897	0,859	998	7,2415	1,1718	0,318	999
**Site x Time**	3	53,202	1,5137	0,158	999	25,671	0,92036	0,494	999	10,014	1,6203	0,205	997
**Residuals**	16	35,148				27,892				6,1801			

		Carbon (r-TCA)				Carbon (CBB)				CCMs				Photosynthesis			
**Source**	**df**	**MS**	**Pseudo-F**	**P(perm)**	**Unique perms**	**MS**	**Pseudo-F**	**P(perm)**	**Unique perms**	**MS**	**Pseudo-F**	**P(perm)**	**Unique perms**	**MS**	**Pseudo-F**	**P(perm)**	**Unique perms**
**Site**	3	831,1	7,0621	**0,001**	998	1765,5	4,6063	**0,012**	999	117,51	4,7023	**0,009**	998	626,06	1,2221	0,238	998
**Time**	1	313,28	2,3102	0,136	998	3075	8,0227	0,016	996	226,68	9,0709	0,003	998	4334,3	8,4606	**0,001**	999
**Site x Time**	3	441,23	2,1358	0,109	998	712,56	1,8591	0,158	999	21,08	0,84357	0,514	999	783,22	1,5288	0,079	998
**Residuals**	16	315,04				383,29				24,989				512,3			
**Pair-wise tests Groups**		**t**	**P(perm)**	**Unique perms**		**t**	**P(perm)**	**Unique perms**		**t**	**P(perm)**	**Unique perms**					
**Vent 1, Vent 2**		2,1597	**0,011**	991		2,0957	**0,076**	990		1,5864	0,126	986					
**Vent 1, REF 1**		1,9704	**0,016**	992		2,8384	**0,026**	994		3,2948	**0,003**	991					
**Vent 1, REF 2**		2,1109	**0,006**	988		2,3601	**0,046**	985		3,2401	**0,008**	988					
**Vent 2, REF 1**		0,78339	0,918	973		1,4608	0,166	979		1,4032	0,17	987					
**Vent 2, REF 2**		0,70692	0,403	988		0,72076	0,462	984		1,1181	0,298	991					
**REF 1, REF 2**		1,0354	0,434	987		0,19254	0,81	991		Negative							
**t1, t2**						2,8324	**0,015**	999		3,0118	**0,006**	998		2,9087	**0,001**	999	

Supplementary Table 9. Permanova analysis at univariate level of sulfur metabolism. Pair-wise test showed comparison between sites (Vent 1, Vent 2, REF 1, REF 2) and times (t1 and t2). Significant results are highlighted. Pair-wise tests were conducted after significant P(perm) results.

		Sulfur			
**Source**	**df**	**MS**	**Pseudo-F**	**P(perm)**	**Unique perms**
**Site**	3	21,902	2,3766	0,01	999
**Time**	1	21,484	2,3313	0,062	999
**Site x Time**	3	20,465	2,2207	**0,019**	999
**Residuals**	16	9,2157			

Pair-wise tests

Within level 't1' of factor 'Time'

**Groups**	**t**	**P(perm)**	**Unique perms**	**P(MC)**
Vent1, Vent2	1,9413	0,076	10	0,066
Vent1, REF1	3,0333	0,106	10	**0,021**
Vent1, REF2	2,6062	0,113	10	**0,045**
Vent2, REF1	1,3825	0,094	10	0,204
Vent2, REF2	1,0094	0,624	10	0,387
REF1, REF2	0,93756	0,491	10	0,463

Within level 't2' of factor 'Time'

**Groups**	**t**	**P(perm)**	**Unique perms**	**P(MC)**
Vent1, Vent2	2,1119	0,092	10	**0,04**
Vent1, REF1	2,6727	0,105	10	**0,023**
Vent1, REF2	1,0167	0,486	10	0,399
Vent2, REF1	2,0992	0,105	10	**0,05**
Vent2, REF2	1,1652	0,216	10	0,274
REF1, REF2	0,91698	0,605	10	0,475

Within level 'Vent1' of factor 'Site'

**Groups**	**t**	**P(perm)**	**Unique perms**	**P(MC)**
t1, t2	2,414	0,101	10	**0,027**

Within level 'Vent2' of factor 'Site'

**Groups**	**t**	**P(perm)**	**Unique perms**	**P(MC)**
Groups				
t1, t2	1,3243	0,103	10	0,206

Within level 'REF1' of factor 'Site'

**Groups**	**t**	**P(perm)**	**Unique perms**	**P(MC)**
t1, t2	3,012	0,103	10	**0,02**

Within level 'REF2' of factor 'Site'

**Groups**	**t**	**P(perm)**	**Unique perms**	**P(MC)**
t1, t2	0,61077	0,889	10	0,705

Supplementary Table 10. SIMPER analysis on predicted abundance of genes for Sulfur metabolism across groups of sites (Vent 1, Vent 2, REF 1, REF 2) and times (t1 and t2).

Sulfur

Groups Vent1 & Vent2

Average dissimilarity = 4,30

	**Group Vent1**	**Group Vent2**				
**Enzyme**	**Av.Abund**	**Av.Abund**	**Av.Diss**	**Diss/SD**	**Contrib%**	**Cum.%**
adenylylsulfate reductase (aprAB) [EC:1.8.99.2]	0,01	0	0,92	0,95	21,38	21,38
sulfate adenylyltransferase (sat) [EC:2.7.7.4]	0,02	0,02	0,72	1,28	16,62	38
sulfide:quinone oxidoreductase [EC:1.8.5.4]	0,02	0,02	0,71	1,61	16,46	54,45
sulfite reductase (NADPH) (dsrAB) [EC:1.8.1.2]	0,02	0,02	0,56	1,21	13	67,46
sulfur-oxidizing protein Sox system (ABXYZ)	0,03	0,03	0,46	1,49	10,61	78,06
sulfur oxygenase/reductase [EC:1.13.11.55]	0	0	0,37	1,6	8,48	86,55
sulfide dehydrogenase [flavocytochrome c] flavoprotein chain [EC:1.8.2.3]	0,01	0,01	0,28	1,31	6,51	93,06

Groups Vent1 & REF1

Average dissimilarity = 4,99

	**Group Vent1**	**Group REF1**			
**Enzyme**	**Av.Abund**	**Av.Abund**	**Av.Diss**	**Diss/SD**	**Contrib%**
adenylylsulfate reductase (aprAB) [EC:1.8.99.2]	0,01	0	1,22	2,57	24,54
sulfate adenylyltransferase (sat) [EC:2.7.7.4]	0,02	0,02	0,85	1,21	17,12
sulfur-oxidizing protein Sox system (ABXYZ)	0,03	0,03	0,68	1,37	13,61
sulfide:quinone oxidoreductase [EC:1.8.5.4]	0,02	0,02	0,57	1,49	11,38
sulfide dehydrogenase [flavocytochrome c] flavoprotein chain [EC:1.8.2.3]	0,01	0,01	0,47	1,35	9,38
sulfur oxygenase/reductase [EC:1.13.11.55]	0	0	0,41	1,81	8,23
sulfite reductase (NADPH) (dsrAB) [EC:1.8.1.2]	0,02	0,02	0,39	1,29	7,89

Groups Vent2 & REF1

Average dissimilarity = 3,97

	Group Vent2	Group REF1				
**Enzyme**	**Av.Abund**	**Av.Abund**	**Av.Diss**	**Diss/SD**	**Contrib%**	**Cum.%**
adenylylsulfate reductase (aprAB) [EC:1.8.99.2]	0	0	1,1	1,61	27,64	27,64
sulfate adenylyltransferase (sat) [EC:2.7.7.4]	0,02	0,02	0,67	1,24	16,86	44,5
sulfite reductase (NADPH) (dsrAB) [EC:1.8.1.2]	0,02	0,02	0,58	1,2	14,52	59,02
sulfur-oxidizing protein Sox system (ABXYZ)	0,03	0,03	0,45	0,86	11,28	70,3
sulfide:quinone oxidoreductase [EC:1.8.5.4]	0,02	0,02	0,42	1,2	10,59	80,89
sulfide dehydrogenase [flavocytochrome c] flavoprotein chain [EC:1.8.2.3]	0,01	0,01	0,41	1,36	10,36	91,25

Groups Vent2 & REF2

Average dissimilarity = 4,82

	Group Vent2	Group REF2			
**Enzyme**	**Av.Abund**	**Av.Abund**	**Av.Diss**	**Diss/SD**	**Contrib%**
adenylylsulfate reductase (aprAB) [EC:1.8.99.2]	0	0	0,92	1,21	19,13
sulfide:quinone oxidoreductase [EC:1.8.5.4]	0,02	0,02	0,84	0,83	17,4
sulfate adenylyltransferase (sat) [EC:2.7.7.4]	0,02	0,02	0,79	0,99	16,45
sulfur-oxidizing protein Sox system (ABXYZ)	0,03	0,03	0,78	1,28	16,14
sulfide dehydrogenase [flavocytochrome c] flavoprotein chain [EC:1.8.2.3]	0,01	0,01	0,55	1,08	11,34
sulfite reductase (NADPH) (dsrAB) [EC:1.8.1.2]	0,02	0,02	0,5	1,25	10,37

Groups Vent1 & REF2

Average dissimilarity = 5,54

	Group Vent1	Group REF2				
**Enzyme**	**Av.Abund**	**Av.Abund**	**Av.Diss**	**Diss/SD**	**Contrib%**	**Cum.%**
adenylylsulfate reductase (aprAB) [EC:1.8.99.2]	0,01	0	1,07	1,44	19,29	19,29
sulfur-oxidizing protein Sox system (ABXYZ)	0,03	0,03	1,04	1,39	18,86	38,15
sulfate adenylyltransferase (sat) [EC:2.7.7.4]	0,02	0,02	0,89	1,16	16,05	54,2
sulfide:quinone oxidoreductase [EC:1.8.5.4]	0,02	0,02	0,83	1,14	14,9	69,1
sulfide dehydrogenase [flavocytochrome c] flavoprotein chain [EC:1.8.2.3]	0,01	0,01	0,59	1,17	10,59	79,69
sulfur oxygenase/reductase [EC:1.13.11.55]	0	0	0,42	1,83	7,58	87,27
polysulfide reductase (psrBC)	0	0	0,35	1,47	6,36	93,62

Groups REF1 & REF2

Average dissimilarity = 4,31

	Group REF1	Group REF2			
**Enzyme**	**Av.Abund**	**Av.Abund**	**Av.Diss**	**Diss/SD**	**Contrib%**
sulfate adenylyltransferase (sat) [EC:2.7.7.4]	0,02	0,02	1,06	1,06	24,55
sulfur-oxidizing protein Sox system (ABXYZ)	0,03	0,03	0,78	1,37	18,12
sulfide:quinone oxidoreductase [EC:1.8.5.4]	0,02	0,02	0,76	0,79	17,7
adenylylsulfate reductase (aprAB) [EC:1.8.99.2]	0	0	0,57	1,05	13,22
sulfide dehydrogenase [flavocytochrome c] flavoprotein chain [EC:1.8.2.3]	0,01	0,01	0,49	1,2	11,33
sulfite reductase (NADPH) (dsrAB) [EC:1.8.1.2]	0,02	0,02	0,35	1,02	8,18

Groups t1 & t2

Average dissimilarity = 4,69

	Group t1	Group t2				
**Enzyme**	**Av.Abund**	**Av.Abund**	**Av.Diss**	**Diss/SD**	**Contrib%**	**Cum.%**
sulfate adenylyltransferase (sat) [EC:2.7.7.4]	0,02	0,02	0,93	1,4	19,91	19,91
adenylylsulfate reductase (aprAB) [EC:1.8.99.2]	0	0	0,85	1,28	18,09	38,01
sulfide:quinone oxidoreductase [EC:1.8.5.4]	0,02	0,02	0,74	1,1	15,86	53,87
sulfur-oxidizing protein Sox system (ABXYZ)	0,03	0,03	0,71	1,31	15,1	68,97
sulfide dehydrogenase [flavocytochrome c] flavoprotein chain [EC:1.8.2.3]	0,01	0,01	0,58	1,69	12,31	81,28
sulfite reductase (NADPH) (dsrAB) [EC:1.8.1.2]	0,02	0,02	0,44	1,3	9,49	90,76

Supplementary Table 11. SIMPER analysis on predicted genes of Photosysnthesis and CCMs metabolic pathways across groups of sites (Vent 1, Vent 2, REF 1, REF 2) and times (t1 and t2).

Photosynthesis

Groups t1 & t2

Average dissimilarity = 34,73

	**Group t1**	**Group t2**				
**Enzyme**	**Av.Abund**	**Av.Abund**	**Av.Diss**	**Diss/SD**	**Contrib%**	**Cum.%**
photosystem II	0,02	0,01	17,26	1,65	49,69	49,69
photosystem I	0,02	0,01	12,07	1,65	34,74	84,44
photosynthetic reaction center	0,02	0,02	5,12	0,64	14,74	99,17

CCMs

Groups Vent1 & Vent2

Average dissimilarity = 8,08

	**Group Vent1**	**Group Vent2**				
**Enzyme**	**Av.Abund**	**Av.Abund**	**Av.Diss**	**Diss/SD**	**Contrib%**	**Cum.%**
carbon dioxide concentrating mechanism protein CcmKLMNO	0	0,01	5,67	1,3	70,22	70,22
carbonic anhydrase [EC:4.2.1.1]	0,02	0,02	2,41	1,33	29,78	100

Groups Vent1 & REF1

Average dissimilarity = 10,92

	**Group Vent1**	**Group REF1**				
**Enzyme**	**Av.Abund**	**Av.Abund**	**Av.Diss**	**Diss/SD**	**Contrib%**	**Cum.%**
carbon dioxide concentrating mechanism protein CcmKLMNO	0	0,01	8,47	1,68	77,52	77,52
carbonic anhydrase [EC:4.2.1.1]	0,02	0,02	2,46	1,23	22,48	100

Groups Vent2 & REF1

Average dissimilarity = 7,77

Enzyme	Group Vent2 Av.Abund	Group REF1 Av.Abund	Av.Diss	Diss/SD	Contrib%	Cum.%
carbon dioxide concentrating mechanism protein CcmKLMNO	0,01	0,01	5,48	1,49	70,55	70,55
carbonic anhydrase [EC:4.2.1.1]	0,02	0,02	2,29	1,2	29,45	100

Groups Vent2 & REF2

Average dissimilarity = 6,95

Enzyme	Group Vent2 Av.Abund	Group REF2 Av.Abund	Av.Diss	Diss/SD	Contrib%	Cum.%
carbon dioxide concentrating mechanism protein CcmKLMNO	0,01	0,01	5,03	1,4	72,37	72,37
carbonic anhydrase [EC:4.2.1.1]	0,02	0,02	1,92	1,15	27,63	100

Groups t1 & t2

Average dissimilarity = 9,24

Species	Group t1 Av.Abund	Group t2 Av.Abund	Av.Diss	Diss/SD	Contrib%	Cum.%
carbon dioxide concentrating mechanism protein CcmKLMNO	0,01	0	6,84	1,7	74	74
carbonic anhydrase [EC:4.2.1.1]	0,02	0,02	2,4	1,32	26	100

Groups Vent1 & REF2

Average dissimilarity = 10,62

Enzyme	Group Vent1 Av.Abund	Group REF2 Av.Abund	Av.Diss	Diss/SD	Contrib%	Cum.%
carbon dioxide concentrating mechanism protein CcmKLMNO	0	0,01	8,07	1,66	76,04	76,04
carbonic anhydrase [EC:4.2.1.1]	0,02	0,02	2,54	1,33	23,96	100

Groups REF1 & REF2

Average dissimilarity = 6,74

Enzyme	Group REF1 Av.Abund	Group REF2 Av.Abund	Av.Diss	Diss/SD	Contrib%	Cum.%
carbon dioxide concentrating mechanism protein CcmKLMNO	0,01	0,01	4,35	1,31	64,6	64,6
carbonic anhydrase [EC:4.2.1.1]	0,02	0,02	2,39	1,22	35,4	100

Supplementary Fig. 1. Taxonomic affiliation of the 16S rRNA transcript sequences. Taxa representing genera that account for at least 1% (on average) of the overall abundance in all samples are shown. A) Phylum-level. B) Class-level.

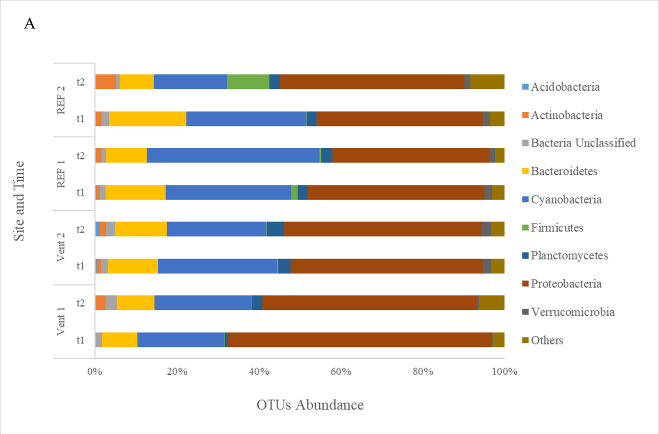

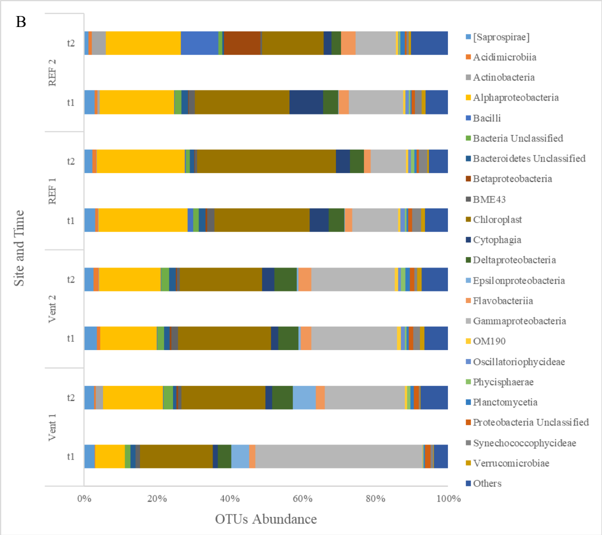

Fig. S1
